# Increased Prevalence of Face Mask—Induced Itch in Health Care Workers

**DOI:** 10.3390/biology9120451

**Published:** 2020-12-07

**Authors:** Piotr K. Krajewski, Łukasz Matusiak, Marta Szepietowska, Rafał Białynicki-Birula, Jacek C. Szepietowski

**Affiliations:** Department of Dermatology, Venereology and Allergology, Wroclaw Medical University, 50-368 Wroclaw, Poland; piotr.krajewski@student.umed.wroc.pl (P.K.K.); luke71@interia.pl (Ł.M.); marta.szepietowska0703@gmail.com (M.S.); rafal.bialynicki-birula@umed.wroc.pl (R.B.-B.)

**Keywords:** personal protective equipment, face masks, health care workers, itch

## Abstract

**Simple Summary:**

The aim of this study was to evaluate the prevalence, intensity and clinical characteristics of face-mask-induced itch during the coronavirus disease 2019 (COVID-19) pandemic in the health care workers (HCW) group. A Google^®^ Forms Internet survey was completed by 1156 HCW. Of the people who wore face masks (three layers of surgical, cloth, respirators and half-face masks), 31.6% reported itch. Sensitive skin, atopic predisposition and facial dermatoses significantly predisposed users to the development of itch. The vast majority of subjects reported itch of moderate intensity. Itch in HCW may cause scratching and decrease the effectiveness of the necessary protection. The results indicate that face-mask-associated itch is an important problem, which should be addressed in future studies. The decreased protection may lead to the spread of the virus among health care workers and their shortage during the pandemic.

**Abstract:**

Background: Face mask use has increased significantly due to the COVID-19 pandemic. Health care workers (HCW) wear masks for prolonged periods and are prone to adverse effects. Very little is known about face-mask-associated itch. Methods: This Internet survey study investigated the prevalence, intensity and clinical characteristics of itch related to the use of face masks by HCW during the COVID-19 pandemic. The results were subsequently compared to the students’ group. Results: A total of 1156 HCW completed the survey. Among them, 31.6% (365) reported suffering from itch associated with face mask use. Itch was more frequent among females. Moreover, subjects who reported sensitive skin, atopic predispositions and facial dermatoses tended to report itch more frequently. The worst case of itch in the seven days prior to the study, assessed with the numeric rating scale (NRS), was 4.6 ± 2.0 points. Itch prevalence increased along with the duration of face mask use, being 34.6% among those who wore masks for more than 4 h. HCW reported itch significantly more frequently than students. Conclusions: Face-mask-associated itch is a frequent problem among HCW in the COVID-19 pandemic. Itch sensation may cause scratching, which may decrease necessary protection during the pandemic.

## 1. Introduction

The first mention about the use of protective face masks in the operating theater was published in 1897 by a Polish surgeon, Jan Mikulicz Radecki, who practiced in Krakow and Wroclaw. It was described as a “mouth bandage” and was a single-layered mask made of gauze [1], which was supposed to protect the patient from wound infection [2]. Face masks began to gain popularity after the First World War; however, many surgeons did not accept them as they were considered “irritating” [1]. Nowadays, health care workers (HCW) use face masks not only during surgery but also to prevent human-to-human respiratory viral transmission [3]. It is believed that the proper use of face masks plays an important role in reducing viral transmission, including the human influenza virus [4], and provides significant protection against the severe acute respiratory syndrome coronavirus (SARS-CoV) [5]. The use of face masks by the general population became a necessity during the SARS (2003) and H1N1 influenza (2009) pandemics [6]. On 11 March, 2020, the World Health Organization announced that the modern SARS-CoV-2, responsible for the coronavirus disease 2019 (COVID-19), should be considered a pandemic [7]. Due to the spring events, the use of face masks has increased drastically not only among HCW but also the general population. More than 50 countries have introduced the obligatory use of face masks covering the mouth and nose in public places [8]. In general, during the ongoing COVID-19 pandemic, face masks have become an everyday clothing accessory. People tend to wear them more frequently and for longer periods.

It has been reported that the prolonged use of personal protective equipment (PPE) may cause adverse skin reactions. The documented adverse effects of face mask use consist of acne, itch, rash, xerosis and nasal bridge scarring [9,10,11].

Itch is the most common symptom in dermatology. It is defined as a sensation leading to scratching and may be a symptom of both dermatological and systemic disorders [12]. Although itch was described as one of the adverse effects of face masks use, little is known about the characteristics and possible risk factor correlations [9]. Due to the lack of sufficient reports and the ongoing development of the pandemic, we decided to conduct a study considering face-mask-induced itch among HCW.

The aim of this study was to assess the prevalence and present the clinical characteristics of face-mask-associated itch among HCW. This study also reports correlations between itch and possible aggravating factors.

## 2. Materials and Methods

### 2.1. Subjects and Study Design

A questionnaire was created and tested among HCW in the Dermatology Department of Wroclaw Medical University. All of the important domains were taken into consideration and used for developing the survey. Subsequently, all questions were evaluated by experts in the itch field (J.C.S. and L.M.). With their comments on correct wording and proper question understanding, the questionnaire was improved. Special attention was given to the itch-related questions. The worst itch associated to face mask use in week prior to the study was assessed with the numeric rating scale (NRS). NRS is a method of itch assessment in which patients assess itch on a scale from 0 to 10, where 0 means no itch and 10 is the worst imaginable itch [13]. The final version of the questionnaire consisted of 17 single-choice questions addressing the following matters: age, sex, workplace and profession; self-reported sensitive skin; present face dermatosis and atopic predispositions; most frequently worn face mask type in and outside of work; presence and severity of itch. The questionnaire was afterward uploaded with the use of Google^®^ Forms and sent to HCW throughout the country. Data were collected in 7 days between 1 October 2020 and 7 October 2020. The survey had to be closed before the introduction of further restrictions in Poland (the introduction of obligatory face mask use for people in public spaces), which would have biased the results. For the control group, a similar technique was used to create an online questionnaire, adjusted for the student population. Analogous questions regarding demographics, self-reported dermatologic problems and attitudes toward mask use were asked. The survey was afterward posted on numerous student groups on social media platforms. These data were collected in the same period.

### 2.2. Statistical Analysis

The results were downloaded for analysis. For responder categorization, according to itch intensity, the following NRS cut-off points were used: 1 to <3 points represented mild itch, 3–7 points represented moderate itch, ≥7 to 9 points represented severe itch and ≥9 points represented very severe itch [13]. A comparison between the control and study groups was performed. The statistical analysis of the obtained results was performed with the use of IBM SPSS Statistics v. 26 (SPSS INC., Chicago, IL, USA) software. All data were assessed for parametric or nonparametric distribution. The minimum, maximum, mean and standard deviation numbers were calculated. Analyzed quantitative variables were evaluated using the Mann–Whitney U test and Spearman’s and Pearson’s correlations, whereas for qualitative data testing, a Chi-squared test was used. Itch intensity among groups was assessed by Kruskal–Wallis one-way analysis of variance on ranks. A two-sided value of *p* ≤ 0.05 was considered statistically significant.

## 3. Results

### 3.1. Group Characteristics

A total of 1156 HCW (211 males (18.3%), 945 females (81.7%)) and 1173 students (297 males (25.3%), 876 females (74.7%)) completed the questionnaire. The mean age in the HCW group was 40.5 ± 11.8 years and 20.9 ± 2.9 years among students. In total, 35.8% (834 people) of the responders reported atopic predispositions. The number was significantly higher among HCW (438 people, 37.9%) than students (396 people, 33.8%) (*p* < 0.001). More than half of the population (51.5%) reported having sensitive skin; however, no differences between groups were found. Statistically significant differences were documented in the presence of dermatosis at the time of completing the survey between students and HCW (57.5% and 37.5%, respectively) (*p* < 0.001). Only 701 people (30.1%) admitted to disinfecting their face masks after use. This procedure was significantly more frequent in the student group (38.3%) (*p* < 0.001) (Table 1).

### 3.2. Itch

Itch was present in 25.8% (602 people) of the participants. Itch prevalence was statistically higher among HCW than students (365 (31.6%) and 237 (20.2%), respectively) (*p* < 0.001). We found a significant difference in itch prevalence between sexes in the whole population (*p* < 0.001), as well as in both studied groups (*p* < 0.001). In all of the groups, females reported itch more frequently than males. The worst itch NRS (WI-NRS) in the last week was 4.6 ± 2.0 points for the whole population, 4.6 ± 2.0 points for HCW and 4.7 ± 2.1 points for students, which indicates a moderate itch severity. No significant difference in itch intensity was found between groups nor sexes (Table 2). The majority of participants reported itch of moderate severity (68.8%), followed by severe (14.5%), mild (14%) and very severe (2.7%). There were no significant differences found between the studied groups. Itch tended to appear more frequently in responders with a self-reported atopic predisposition, sensitive skin and present dermatosis. In all of the mentioned cases, the HCW group reported itch more frequently than the student group (Table 3). Itch prevalence tended to grow along with the duration of face mask use. Wearing a mask caused itch in 19.6% of participants who wore masks up to one hour and 33.2% of participants who wore masks for longer periods (*p* < 0.001 for the cut-off point of 4 h). This was shown only for the whole population; however, there were no differences reported for the separate groups (Table 4). Moreover, there was no difference in itch severity in relation to the duration of face mask use. With regards to the type of face mask used most frequently, itch was most common in those wearing respirators (N95/FFP2) (32.6%), whereas cloth masks caused itch only in 20% of users (*p* < 0.001) (Table 5). Finally, there was no difference regarding itch prevalence in the separate analysis of students and HCW.

## 4. Discussion

Personal protective equipment is commonly used among workers in hazardous environments [14]. Due to the significantly increased occupational risk, it is also among the basic equipment for HCW working with high-risk patients (e.g., Ebola virus and Middle Eastern respiratory syndrome coronavirus patients) [15,16]. The COVID-19 pandemic has caused a drastic change in the use of PPE among HCW. New guidelines for numerous medical specializations have been introduced [17,18,19]. Complete PPE for working with patients with COVID-19 includes an N95 respirator, a head covering, eye protection, a gown and a single pair of gloves [19]. However, according to the WHO guidelines regarding the rational use of PPE for COVID-19 [20], complete gear should be used only for those working directly with patients with COVID-19. Due to the severe shortage of PPE, other medical staff should use face masks of any type in health care settings [20]. It is important to emphasize that prolonged use of PPE may cause adverse effects and discomfort. According to the available studies, up to 62% of workers reported PPE to be uncomfortable [14,21]. The most frequently used part of full COVID-19 PPE is the face mask. Currently, people tend to wear cloth and surgical masks rather than professional respirators (N95/FFP2), half-face respirators and full-face masks [22]. Among the possible adverse effects of prolonged face mask use, authors frequently mention xerosis, rash, acne, facial dermatitis, pigmentation of the nasal bridge, chicks or chin and itching [10,11,23,24].

Itch is the most common symptom in dermatology. According to the International Forum for the Study of Itch, it is defined as a sensation that provokes the desire to scratch [25]. The prevalence of itch is not clear; however, it is believed that about 8–9% of the adult population experience acute itch and 16.8% experience chronic itch [26]. Itch may be caused by both dermatological and systemic disorders [12]. It is important to emphasize that itch has a well documented negative effect on patients’ quality of life [27]. Itch in the era of the COVID-19 pandemic was analyzed by our group earlier this year [28]. Available reports suggest that COVID-19-associated dermatoses (e.g., urticaria, erythematous rash or varioliform eruptions) may be accompanied by itch; however to the best of our knowledge, there are no sufficient data available to fully characterize this [28]. Furthermore, itch may be caused by other pandemic-associated factors, such as the use of cleaning chemicals, psychosocial stress and prolonged use of PPE. It is well documented that anxiety levels during the COVID-19 pandemic have increased among the general population [29,30]. Moreover, it is known that stress and fear may cause or aggravate itch sensation [31]. Similarly, proper hand hygiene and excessive use of hand disinfectants or gloves may lead to the development of hand eczemas [32]. According to available reports, up to 74% of HCW admitted to hand and skin damage [33]. The itch sensation was not studied directly; however, skin dryness, reported in up to 68.6% of subjects [34], is a leading cause of itch in unchanged skin [35].

The presence of face-mask-associated itch is not sufficiently described. Authors mention itch among other adverse effects and assess its prevalence at 15.6–51.4% [10,11,36]. To the best of our knowledge, this is the second study directly addressing the topic of face-mask-induced itch. The first study was conducted by our group at the beginning of the pandemic and assessed self-reported itch among young people wearing masks [9]. In the current study, about 25.8% of the responders reported itch in the week prior to the study, and the number was significantly higher for the HCW (31.6%) than for students (20.2%). Our results are in agreement with our previous study, in which about 20% of the responders reported itch. The difference between students and HCW in both studies/young people may be caused by several factors. Primarily, studies with higher itch incidence (51.4% and 27.9%) [10,11] concentrated mostly on HCW rather than the general population. It is well known and was demonstrated in our study that HCW tend to wear professional equipment (e.g., respirators) rather than cloth masks. The type of face mask is directly associated with the increasing prevalence of itch in responders of our study (Table 5). Moreover, due to the excessive exposure and care of high-risk patients, medical personnel use face masks for much longer periods. Among the general population, face masks are currently worn only in public spaces and are limited for essential activities in closed spaces. As previously demonstrated by Szepietowski et al. [9], itch is more common in groups of responders using face masks for 5 h and longer. Our data stay in agreement with those results, as itch incidence increased along with the duration of face mask use.

The current study documents a difference in itch sensation between subjects with self-reported sensitive skin, atopy and present facial dermatosis. Sensitive skin is a subjective term that refers to patient observations regarding burning, stinging, itch and tightness due to environmental stimuli [37]. Although the reason some subjects are more prone to itching is still unclear, we reported a significantly higher number of people with sensitive skin suffering from face-mask-induced itch. This may be provoked by the degranulation of mast cells triggered by stress associated both with the ongoing pandemic situation and prolonged face mask use [37] or abnormal itching from thermal stimuli [38]. It is well documented that people with an atopic predisposition and atopic dermatitis suffer frequently from itch [38,39]. Similarly, our responders with an atopic predisposition tended to have a higher risk of itch development than others. Furthermore, the risk of face-mask-induced itch was significantly higher in people with present facial dermatoses (e.g., acne or seborrheic dermatitis). The mentioned diseases are often associated with itch [40,41]. It is important to emphasize that prolonged use of face masks may also aggravate dermatosis and therefore increase itch sensation, which was proven in the study by Zuo et al. [42], who evaluated skin reaction to masks among Chinese HCW. Authors have observed an exacerbation in 44.2% of patients, mostly with acne, seborrheic dermatitis and rosacea [42]. Interestingly, there was a difference in itch prevalence between HCW and students with present facial dermatosis. Although more students reported suffering from facial skin problems at the time of survey completion, it was the HCW group who reported itch more frequently. This may be caused by age differences and different dermatological conditions. Acne, which is the most common among young subjects, may be the origin of itch in about 14% of the subjects [40], whereas in rosacea, the percentage of patients suffering from itch is significantly higher (up to 70%) [43].

We found that face-mask-induced itch was of moderate severity (WI-NRS 4.6 ± 2 points) and the majority of responders (66.6%) experienced moderate itch. Our results are similar to those presented by Szepietowski et al. [9], who reported itch of similar intensity among young people. We did not find any difference in itch prevalence between the studied groups. Women in all groups tended to suffer more frequently from face-mask-associated itch. Sex differences in itch perception were previously studied by Ständer et al. [44]. The authors reported that, in comparison to men, women suffer from higher itch intensity with a larger impact on their quality of life [44]. However, there was no statistically significant difference in itch intensity regarding sex found in this study.

We understand that the study has some limitations. An online survey does not allow conducting a meticulous dermatological examination with a proper medical history. Unfortunately, due to the COVID-19 pandemic, face-to-face encounters with hundreds of subjects were not possible; thus, atopic predisposition, sensitive skin and present facial dermatosis were self-reported. Furthermore, we understand that it is impossible to assess the response rate with this methodology. Regardless, during the COVID-19 pandemic, it has become a frequently used method for this type of study [45,46]. We are aware that our control and the studied groups are not consistent regarding age. However, according to the performed age-adjusted analysis, age was not a predictor for face-mask-associated itch occurrence (*p* > 0.05). Moreover, several types of material might be used to manufacture face masks. Because of such differences, we could not establish a direct connection to the used material but only to the face mask type.

## 5. Conclusions

In conclusion, the COVID-19 pandemic has drastically changed everyday life. It has resulted in several new practices, such as face mask use in public spaces or the continuous necessity of HCW to wear face protection. This study demonstrates that HCW who wear face masks for prolonged periods may suffer from itch more frequently than casual mask users. The itch sensation may lead to scratching or touching the face masks, which may compromise the effectiveness of their protection. Due to the further development of the COVID-19 pandemic and an obligation to wear masks, future original studies are necessary to discover the possible management of face-mask-associated itch.

## Figures and Tables

**Table 1:** Characteristics.

Characteristics		Population (*n* = 2329)	HCW (*n* = 1156)	Students (*n* = 1173)	*p*
**Sex.** *n* (%)	Female	1821	945	876	**<0.001**
		(78.2%)	(81.7%)	(74.7%)	
	Male	508	211	297	
		(21.8%)	(18.3%)	(25.3%)	
Age. Mean ± SD (years)		30.6 ± 13.0	40.5 ± 11.8	20.9 ± 2.9	**<0.001**
Atopy		834	438	396	**0.038**
*n* (%)		(35.8%)	(37.9%)	(33.8%)	
Sensitive skin		1199	586	613	0.449
*n* (%)		(51.5%)	(50.7%)	(52.3%)	
Facial dermatosis		1107	433	674	**<0.001**
*n* (%)		(47.5%)	(37.5%)	(57.5%)	
Disinfection		701	252	449	**<0.001**
*n* (%)		(30.1%)	(21.8%)	(38.3%)	

HCW—health care workers; bold—*p* < 0.05*; n*—number of participants; SD—standard deviation.

**Table 2:** Characteristics.

Characteristics		Population (*n* = 2329)	HCW (*n* = 1156)	Students (*n* = 1173)	*p*
**Itch** *n* (%)	Whole group	602	**365**	237	<0.001
		(25.8%)	**(** **31.6%)**	(20.2%)	
	Female	520 *	320.0 *	200 *	0.007
		(28.6%)	**(33.9** **%)**	(22.8%)	
	Male	82 *	45.0 *	37 *	<0.001
		(16.1%)	(21.3%)	(12.5%)	
Previous week WI-NRS Mean ± SD (points)	Whole group	4.6 ± 2	4.6 ± 2	4.7 ± 2.1	0.758
	Female	4.6 ± 2.0	4.6 ± 1.9	4.6 ± 2.0	0.957
	Male	4.9 ± 2.3	4.7 ± 2.2	5.1 ± 2.3	0.420

HCW—health care workers; bold—*p* < 0.05*; n*—number of participants; WI-NRS—worst itch numeric rating scale; asterisk (*)—statistical difference between sexes (*p* < 0.005); SD—standard deviation.

**Table 3:** Predisposing factors.

	HCW (*n* = 1156)			Students (*n* = 1173)		p*	
	Population	Itch	*p*	Population	Itch	*p*	
Atopy	Yes	159 *	**0.007**	Yes	96 *	**0.014**	**<0.001**
	(*n* = 438)	(36.3%)		(*n* = 396)	(24.2%)		
	No	206		No	141*		
	(*n* = 718)	(28.7%)		(*n* = 777)	(18.1%)		
Sensitive skin	Yes	247 *	**<0.001**	Yes	174 *	**<0.001**	**<0.001**
	(*n* = 586)	(42.2%)		(*n* = 613)	(28.4%)		
	No	118		No	63		
	(*n* = 570)	(20.7%)		(*n* = 560)	(11.3%)		
Facial Dermatosis	Yes	214 *	**<0.001**	Yes	171*	**<0.001**	**<0.001**
	(*n* = 433)	(49.4%)		(*n* = 674)	(25.4%)		
	No	151		No	66		
	(*n* = 723)	(20.9%)		(*n* = 499)	(13.2%)		
Disinfection	Yes	87	0.255	Yes	98	0.276	**<0.001**
	(*n* = 252)	(34.5%)		(*n* = 449)	(21.8%)		
	No	278		No	139		
	(*n* = 904)	(30.8%)		(*n* = 724)	(19.2%)		

HCW—health care workers; bold—*p* < 0.05; *n*—number of participants; p*—statistical difference between two groups; asterisk (*)—statistical difference between sexes (*p* < 0.005).

**Table 4:** Severity in relation to the duration of face mask use.

Time	Population (*n* = 2329)	Itch (*n* = 602)	No Itch (*n* = 1727)	*p*
Up to 1 h	Whole	125	513	**<0.001**
	(*n* = 638)	(20.8%)	(29.7%)	
	HCW	2	13	0.126
	(*n* = 15)	(0.5%)	(1.6%)	
	Students	123	500	0.675
	(*n* = 623)	(51.9%)	(53.4%)	
Up to 2 h	Whole	229	872	**<0.001**
	(*n* = 1101)	(38.0%)	(50.4%)	
	HCW	22	62	0.270
	(*n* = 84)	(6.0%)	(7.8%)	
	Students	207	810	0.745
	(*n* = 1017)	(87.3%)	(86.5%)	
Up to 4 h	Whole	286	1089	**<0.001**
	(*n* = 1375)	(47.5%)	(63.1%)	
	HCW	61	215	**<0.001**
	(*n* = 276)	(16.7%)	(27.2%)	
	Students	225	874	0.377
	(*n* = 1099)	(94.9%)	(93.4%)	
More than 4 h	Whole	316	637	**<0.001**
	(*n* = 953)	(52.5%)	(36.9%)	
	HCW	304	575	**<0.001**
	(*n* = 879)	(83.3%)	(72.7%)	
	Students	12	62	0.377
	(*n* = 74)	(5.1%)	(6.6%)	

HCW—health care workers; bold—*p* < 0.05; *n*—number of participants.

**Table 5:** Prevalence difference according to face mask types.

Face Mask Type	Population	Itch	No Itch	*p* (Compared to Other Types)
Surgical	Whole (*n* = 1363)	367	996	0.158
		(26.9%)	(73.1%)	
	HCW	248	563	0.265
	(*n* = 811)	(30.6%)	(69.4%)	
	Students	119	433	0.276
	(*n* = 552)	(21.6%)	(78.4%)	
Cloth	Whole	118	473	**<0.001**
	(*n* = 591)	(20%)	(80%)	
	HCW	11	20	0.635
	(*n* = 31)	(35.5%)	(64.5%)	
	Students	107	453	0.371
	(*n* = 560)	(19.1%)	(80.9%)	
Respirator	Whole	72	149	**0.016**
	(*n* = 221)	(32.6%)	(67.4%)	
	HCW	65	119	0.232
	(*n* = 184)	(35.5%)	(64.7%)	
	Students	7	30	0.843
	(*n* = 37)	(18.9%)	(81.1%)	
Half-face	Whole	43	95	0.142
	(*n* = 138)	(31.2%)	(68.8%)	
	HCW	41	87	0.906
	(*n* = 128)	(32%)	(68%)	
	Students	2	8	0.987
	(*n* = 10)	(20%)	(80%)	

HCW—health care workers; bold—*p* < 0.05; *n*—number of participants.
